# Supplementary material for: Efficient processing of complex XSD using Hive and Spark
Source: PeerJ Comput Sci. 2021 Aug 17;7:e652. doi: 10.7717/peerj-cs.652 (PMC8384044; doi:10.7717/peerj-cs.652)
Supplement: Supplemental Information 5 — The file extracts the measurement collection data file XML schema for performance management files in mobile networks in A.4.2 section, and the mapping of ASN.1 Measurement Report File Format tags to XML tags Table A.1, from the 3GG TS 32.401 standard. [file peerj-cs-07-652-s005.docx]

A.4 XML schema based XML file format definition

## A.4.2 Measurement collection data file XML schema

The following XML schema measCollec.xsd is the schema for measurement collection data XML files:

<?xml version="1.0" encoding="UTF-8"?>

<!--
 3GPP TS 32.401 PM Concept and Requirements
 Measurement collection data file XML schema
 measCollec.xsd
-->

<schema
 targetNamespace=
"http://www.3gpp.org/ftp/specs/latest/rel-5/32_series/32401-540.zip#measCollec"
 elementFormDefault="qualified"
 xmlns="http://www.w3.org/2001/XMLSchema"
 xmlns:mc=
"http://www.3gpp.org/ftp/specs/latest/rel-5/32_series/32401-540.zip#measCollec"
>

 <!-- Measurement collection data file root XML element -->

 <element name="measCollecFile">
 <complexType>
 <sequence>
 <element name="fileHeader">
 <complexType>
 <sequence>
 <element name="fileSender">
 <complexType>
 <attribute name="localDn"
 <attribute name="elementType" type="string" use="optional"/>
 </complexType>
 </element>
 <element name="measCollec">
 <complexType>
 <attribute name="beginTime" type="dateTime" use="required"/>
 </complexType>
 </element>
 </sequence>
 <attribute name="fileFormatVersion" type="string" use="required"/>
 <attribute name="vendorName" type="string" use="optional"/>
 <attribute name="dnPrefix" type="string" use="optional"/>
 </complexType>
 </element>
 <element name="measData" minOccurs="0" maxOccurs="unbounded">
 <complexType>
 <sequence>
 <element name="managedElement">
 <complexType>
 <attribute name="localDn" type="string" use="optional"/>
 <attribute name="userLabel" type="string" use="optional"/>
 <attribute name="swVersion" type="string" use="optional"/>
 </complexType>
 </element>
 <element name="measInfo" minOccurs="0" maxOccurs="unbounded">
 <complexType>
 <sequence>
 <element name="granPeriod">
 <complexType>
 <attribute
 name="duration"
 type="duration"
 use="required"
 />
 <attribute
 name="endTime"
 type="dateTime"
 use="required"
 />
 </complexType>
 </element>
 <choice>
 <element name="measTypes">
 <simpleType>
 <list itemType="Name"/>
 </simpleType>
 </element>
 <element name="measType"
 minOccurs="0" maxOccurs="unbounded">
 <complexType>
 <simpleContent>
 <extension base="Name">
 <attribute name="p"
 type="positiveInteger" use="required"/>
 </extension>
 </simpleContent>
 </complexType>
 </element>
 </choice>
 <element name="measValue"
 minOccurs="0" maxOccurs="unbounded">
 <complexType>
 <sequence>
 <choice>
 <element name="measResults">
 <simpleType>
 <list itemType="mc:measResultType"/>
 </simpleType>
 </element>
 <element name="r"
 minOccurs="0" maxOccurs="unbounded">
 <complexType>
 <simpleContent>
 <extension base="mc:measResultType">
 <attribute name="p" type="positiveInteger"
 use="required"/>
 </extension>
 </simpleContent>
 </complexType>
 </element>
 </choice>
 <element name="suspect" type="boolean" minOccurs="0"/>
 </sequence>
 <attribute name="measObjLdn"
 type="string" use="required"/>
 </complexType>
 </element>
 </sequence>
 </complexType>
 </element>
 </sequence>
 </complexType>
 </element>
 <element name="fileFooter">
 <complexType>
 <sequence>
 <element name="measCollec">
 <complexType>
 <attribute name="endTime" type="dateTime" use="required"/>
 </complexType>
 </element>
 </sequence>
 </complexType>
 </element>
 </sequence>
 </complexType>
 </element>

 <simpleType name="measResultType">
 <union memberTypes="decimal">
 <simpleType>
 <restriction base="string">
 <enumeration value="NIL"/>
 </restriction>
 </simpleType>
 </union>
 </simpleType>

</schema>

**Table A.1 Mapping of ASN.1 Measurement Report File Format tags to XML tags**

| **ASN.1 Tag** | **DTD based XML**  **tag** | **XML schema based XML tag** | **Description** |
| --- | --- | --- | --- |
| MeasDataCollection | mdc | measCollecFile | This is the top-level tag, which identifies the file as a collection of measurement data. The file content is made up of a header ("measFileHeader"), the collection of measurement result items ("measData"), and a measurement file footer ("measFileFooter"). |
| measFileHeader | mfh | fileHeader | This is the measurement result file header to be inserted in each file. It includes a version indicator, the name, type and vendor name of the sending network node, and a time stamp ("collectionBeginTime"). |
| measData | md | measData | The "measData" construct represents the sequence of zero or more measurement result items contained in the file. It can be empty in case no measurement data can be provided. The individual "measData" elements can appear in any order.  Each "measData" element contains the name of the NE ("nEId") and the list of measurement results pertaining to that NE ("measInfo"). |
| measFileFooter | mff | fileFooter | The measurement result file footer to be inserted in each file. It includes a time stamp, which refers to the end of the overall measurement collection interval that is covered by the collected measurement results being stored in this file. |
| fileFormatVersion | ffv | fileHeader fileFormatVersion | This parameter identifies the file format version applied by the sender. The format version defined in the present document shall be the abridged number and version of this 3GPP document (see below) for XML formats and the ASN.1 format alike.  The abridged number and version of a 3GPP document is constructed from its version specific full reference "3GPP […] (yyyy-mm)" by:  - removing the leading "3GPP TS"  - removing everything including and after the version third digit, representing editorial only changes, together with its preceding dot character  - from the resulting string, removing leading and trailing white space, replacing every multi character white space by a single space character and changing the case of all characters to uppercase. |
| senderName | sn | fileHeader dnPrefix  and fileSender localDn | The senderName uniquely identifies the NE or EM that assembled this measurement file by its Distinguished Name (DN), according to the definitions in 3GPP TS 32.300 [10]. In the case of the NE-based approach, it is identical to the sender's "nEDistinguishedName".  For ASN.1 and DTD based XML format, the string may be empty (i.e. string size =0) in case the DN is not configured in the sender.  For the XML schema based XML format, the DN is split into the DN prefix and the Local DN (LDN) (see 3GPP TS 32.300 [10]). XML attribute specification "dnPrefix" may be absent in case the DN prefix is not configured in the sender. XML attribute specification "localDn" may be absent in case the LDN is not configured in the sender. |
| senderType | st | fileSender elementType | This is a user configurable identifier of the type of network node that generated the file, e.g. NodeB, EM, SGSN. The string may be empty (i.e. string size =0) in case the "senderType" is not configured in the sender.  For the XML schema based XML format, XML attribute specification "elementType" may be absent in case the "senderType" is not configured in the sender. |
| vendorName | vn | fileHeader vendorName | The "vendorName" identifies the vendor of the equipment that provided the measurement file. The string may be empty (i.e. string size =0) if the "vendorName" is not configured in the sender.  For the XML schema based XML format, XML attribute specification "vendorName" may be absent in case the "vendorName" is not configured in the sender. |
| collectionBeginTime | cbt | measCollec beginTime | The "collectionBeginTime" is a time stamp that refers to the start of the first measurement collection interval (granularity period) that is covered by the collected measurement results that are stored in this file. |
| nEId | neid | managedElement | The unique identification of the NE in the system. It includes the user name ("nEUserName"), the distinguished name ("nEDistinguishedName") and the software version ("nESoftwareVersion") of the NE. |
| nEUserName | neun | managedElement userLabel | This is the user definable name ("userLabel") defined for the NE in 3GPP TS 32.622 [24]. The string may be empty (i.e. string size =0) if the "nEUserName" is not configured in the CM applications.  For the XML schema based XML format, XML attribute specification "userLabel" may be absent in case the "nEUserName" is not configured in the CM applications. |
| nEDistinguishedName | nedn | fileHeader dnPrefix  and managedElement localDn | This is the Distinguished Name (DN) defined for the NE in 3GPP TS 32.300 [10]. It is unique across an operator's 3G network. The string may be empty (i.e. string size =0) if the "nEDistinguishedName" is not configured in the CM applications.  For the XML schema based XML format, the DN is split into the DN prefix and the Local DN (LDN) (see 3GPP TS 32.300 [10]). XML attribute specification "localDn" may be absent in case the LDN is not configured in the CM applications. |
| nESoftwareVersion | nesw | managedElement swVersion | This is the software version ("swVersion") defined for the NE in 3GPP TS 32.622 [24].  This is an optional parameter which allows post-processing systems to take care of vendor specific measurements modified between software versions.  For the XML schema based XML format, XML attribute specification "swVersion" may be absent in case the "nESoftwareVersion" is not configured in the CM applications. |
| measInfo | mi | measInfo | The sequence of measurements, values and related information. It includes a list of measurement types ("measTypes") and the corresponding results ("measValues"), together with the time stamp ("measTimeStamp") and granularity period ("granularityPeriod") pertaining to these measurements. |
| measTimeStamp | mts | granPeriod endTime | Time stamp referring to the end of the granularity period. |
| granularityPeriod | gp | granPeriod duration | Granularity period of the measurement(s) in seconds.  For the XML schema based XML format, the value of XML attribute specification "duration" shall use the truncated representation "PT*n*S" (see [28]). |
| measTypes | mt | measTypes  or measType | This is the list of measurement types for which the following, analogous list of measurement values ("measValues") pertains. The GSM only measurement types are defined in TS 52.402 [22]. The measurement types for UMTS and combined UMTS/GSM implementations are specified in TS 32.403 [23].  For the XML schema based XML format, depending on sender's choice for optional positioning presence, either XML element "measTypes" or XML elements "measType" will be used. |
| measValues | mv | measValue | This parameter contains the list of measurement results for the resource being measured, e.g. trunk, cell. It includes an identifier of the resource ("measObjInstId"), the list of measurement result values ("measResults") and a flag that indicates whether the data is reliable ("suspectFlag"). |
| measObjInstId | moid | measValue measObjLdn | The "measObjInstId" field contains the local distinguished name (LDN) of the measured object within the scope defined by the "nEDistinguishedName" (see 3GPP TS 32.300 [10]). The concatenation of the "nEDistinguishedName" and the "measObjInstId" yields the DN of the measured object. The "measObjInstId" is therefore empty if the "nEDistinguishedName" already specifies completely the DN of the measured object, which is the case for all measurements specified on NE level. For example, if the measured object is a "ManagedElement" representing RNC "RNC-Gbg-1", then the "nEDistinguishedName" will be for instance "DC=a1.companyNN.com,SubNetwork=1,IRPAgent=1,SubNetwork=CountryNN,MeContext=MEC-Gbg-1,ManagedElement=RNC-Gbg-1", and the "measObjInstId" will be empty. On the other hand, if the measured object is a "UtranCell" representing cell "Gbg-997" managed by that RNC, then the "nEDistinguishedName" will be for instance the same as above, i.e. "DC=a1.companyNN.com,SubNetwork=1,IRPAgent=1,SubNetwork=CountryNN,MeContext=MEC-Gbg-1,ManagedElement=RNC-Gbg-1", and the "measObjInstId" will be for instance "RncFunction=RF-1,UtranCell=Gbg-997". The class of the "measObjInstId" is defined in item F of each measurement definition template. |
| measResults | r | measResults  or  r | This parameter contains the sequence of result values for the observed measurement types. The "measResults" sequence shall have the same number of elements, which follow the same order as the measTypes sequence. Normal values are INTEGERs and REALs. The NULL value is reserved to indicate that the measurement item is not applicable or could not be retrieved for the object instance.  For the XML schema based XML format, depending on sender's choice for optional positioning presence, either XML element "measResults" or XML elements "r" will be used. |
| suspectFlag | sf | suspect | Used as an indication of quality of the scanned data. FALSE in the case of reliable data, TRUE if not reliable. The default value is "FALSE", in case the suspect flag has its default value it may be omitted. |
| timeStamp | ts | measCollec endTime | This tag carries the time stamp that refers to the end of the measurement collection interval (granularity period) that is covered by the collected measurement results that are stored in this file. The minimum required information within timestamp is year, month, day, hour, minute, and second. |
| Not Required | mt p | measType p | An optional positioning XML attribute specification of XML elements "mt" (DTD based) and "measType" (XML schema based), used to identify a measurement type for the purpose of correlation to a result. The value of this XML attribute specification is expected to be a non-zero, non-negative integer value that is unique for each instance of XML element "mt" or "measType" that is contained within the measurement data collection file. |
| Not Required | r p | r p | An optional positioning XML attribute specification of XML element "r", used to correlate a result to a measurement type. The value of this XML attribute specification should match the value of XML attribute specification "p" of corresponding XML element "mt" " (DTD based) or "measType" (XML schema based). |
